# Transplantation of Human Umbilical Cord Blood-Derived Mesenchymal Stem Cells Improves Cartilage Repair in a Rabbit Model

**DOI:** 10.1155/2021/6380141

**Published:** 2021-02-25

**Authors:** Guihua Yang, Jiashen Shao, Jiachen Lin, Haixia Yang, Jing Jin, Chenxi Yu, Bo Shen, Xiaorui Hu, Huijie Si, Xiaoxin Li, Yuchen Niu, Zhihong Wu

**Affiliations:** ^1^Harmony Technology Co., Ltd., Beijing, China; ^2^Department of Orthopedic Surgery, Chinese Academy of Medical College & Peking Union Medical College Hospital, Beijing, China; ^3^Medical Research Center, Chinese Academy of Medical College & Peking Union Medical College Hospital, Beijing, China

## Abstract

The aim of this study was to investigate the therapeutic efficacy and safety of transplanting human umbilical cord blood-derived mesenchymal stem cells (hUCB-MSCs) in the treatment of cartilage injury. First, the articular cartilage defect model in rabbits was constructed. Then, the identified hUCB-MSCs and rabbit bone marrow stem cells (rBM-MSCs) were transplanted into the bone defect, respectively, and the cartilage repair effect was observed by hematoxylin-eosin (HE) staining and immunohistochemistry. Besides, the glycosaminoglycan (GAG) content and biomechanics of the restoration area were also evaluated. In our study, hUCB-MSCs and rBM-MSCs exhibited typical MSC characteristics, with positive expressions of CD73, CD105, and CD90 and negative for CD45, CD34, CD14, and HLA-DR. After the transplantation of hUCB-MSCs and rBM-MSCs, the overall quality of cartilage tissue was significantly improved, and the recipients did not show significant side effects in general. However, the expression of matrix metalloproteinase-13 (MMP-13) in the de novo tissues of the hUCB-MSCs and rBM-MSCs groups was both increased, indicating that the novel tissues may have some potential osteoarthritic changes. In conclusion, our results suggest the therapeutic effect of hUCB-MSCs transplantation in cartilage regeneration, providing a promising future in the clinical treatment of cartilage injury.

## 1. Introduction

Osteoarthritis (OA) is a chronic disorder of degenerative joints involving mostly weight-bearing joints such as the knees and hips. While articular cartilage defects are a common problem in orthopedic surgery and can lead to OA [1]. Cartilaginous chondrocytes are mainly supplied by synovial fluid and the surrounding extracellular matrix, with limited self-repair capacity. Moreover, the avascular environment in the articular cartilage region provides no potency for fibrotic formation and inflammatory cell migration, making it hard for the injured cartilage tissue to regenerate itself during osteoarthritis development, and the quality of the repaired tissue from which is still far from ideal [2, 3]. The therapeutic approach to achieve significant and consistent articular cartilage regeneration under certain disease conditions is still an unmet clinical need.

Clinically, the goal of articular injury treatment is to improve cartilage repair by restoring mechanically functional cartilage tissue [4]. Cartilage tissue engineering has been aiming to restore the hyaline cartilage tissue similar to the physiological conditions. Mesenchyme is derived from mesoderm cells during the embryonic period. Mesenchymal consists of mesenchymal cells and an amorphous matrix. Recently, the development of regenerative medicine has revealed a promising future of applying mesenchymal stem cells (MSCs) in cartilage tissue engineering, due to their self-renewal capacity, multilineage differentiation potential, and immunomodulatory ability [5]. Bone marrow stem cells (BMSC) are the most common source of MSCs and were found to promote cartilage regeneration in vivo. However, the harvest of BMSCs from human donors is invasive [6, 7]. In contrast, human umbilical cord blood-derived MSCs (hUCB-MSCs) was investigated recently as a new source of MSCs considering they are clinically available, preservable with great proliferative capacity in vitro [8]. Additionally, some research data showed that hUCB-MSCs also function as immune regulators with nursing effects during inflammatory responses [9].

To date, although several studies have demonstrated the chondrogenic potential of hUCB-MSCs, the preclinical reports are limited and with inconsistent results [10, 11]. Herein, the aim of the present study was to investigate the therapeutic effect of hUC-MSCs transplanting to a rabbit surgical model compared to allogenic BMSCs, and to verify the regenerative efficacy, consistency, and safety of hUCB-MSC transplantation in the treatment of cartilage injury.

## 2. Materials and Methods

### 2.1. Isolation and Culture of hUCB-MSCs

Human umbilical blood (hUCB) was collected from the neonatal umbilical veins immediately after delivery by an independent umbilical cord blood bank, with informed consent signed by the parents of the donors. After the collection of hUCB (50-100 ml), the isolation and cultivation of the MSCs from the UCB were performed according to the published method [3]. Mononuclear cells were separated via 300 g centrifuge at room temperature and cultured in *α*-minimum essential medium (a-MEM, Gibco BRL, Carlsbad, CA, USA), supplemented with 15% fetal bovine serum (FBS; HyClone, Logan, UT, USA) and then incubated at 37°C in a humidified atmosphere of 5% CO_2_. After two weeks, the nonadherent cells were removed by medium change. The plastic-adherent cells were passaged after confluence using 0.05% trypsin (HyClone, Logan, UT, USA) and continuously subcultured. All hUCB-MSCs used were at passage 6 [3].

### 2.2. Isolation and Culture of rBM-MSCs

New Zealand pure-bred white rabbits weighing 3.0-4.0 kg with large ears were selected. Intravenous anesthesia with 3 g/L pentobarbital sodium (1 ml/kg) at the ear margin was performed. After skin preparation and sterilization of the posterior superior iliac crest, a No. 16 bone marrow puncture needle (Shanghai Poly Medical Instruments Co., LTD, Shanghai, China) with diluted heparin sodium was used to extract about 5.0 ml of bone marrow aspirate. rBM-MSCs were isolated and cultured the same as the method described above.

### 2.3. Flow Cytometry

After trypsinization and resuspension in a blocking buffer containing Hank's balanced salt solution supplemented with 1% BSA for 30 min, the hUCB-MSCs and rBM-MSCs were prepared as single-cell suspensions. Approximately, 1 × 10^6^ cells/ml was incubated with CD105, CD73, CD34, CD45, CD14, and HLA-DR for 45 minutes at 4°C in the dark. After washing three times with PBS, cells were fixed in fluorescence-activated cell sorting fix solution and then analyzed using a Beckman Coulter flow cytometer and FACScan program Cytomics (BD Biosciences, USA).

### 2.4. Animals and Groups

New Zealand white rabbit weighing 1.5 to 2 kg were randomly divided into four groups: control group (*n* = 8), model group (cartilage was surgically damaged followed by normal saline treatment, *n* = 8), hUCB-MSC group (cartilage was surgically damaged followed by hUCB-MSC treatment, *n* = 8), and rBM-MSC group (cartilage was surgically damaged followed by rBM-MSC treatment, *n* = 8). This study was approved by the Ethics Committee of China. All experiments were carried out in accordance with the National Institute of Health Guide for the Care and Use of Laboratory Animals.

### 2.5. Cartilage Injury Model

The articular cartilage injury was generated following a previous study [12]. Briefly, the rabbits were anesthetized first. Both knee joints were draped sterilely and opened via a medial parapatellar approach, and the patella was dislocated laterally. Full-thickness osteochondral defects were created in the trochlear groove of the femur by careful drilling in a vertical direction. After removing the cartilage and bone debris, the boundaries of the drilled holes were trimmed, and the defect sites were carefully washed using saline. Then, 1.5-ml hUCB-MSC and rBM-MSC (6 × 10^6^) solution was transplanted into the injured area, respectively. After transplantation, the patella was relocated, and the soft tissue was closed in layers. All rabbits were allowed to move their knee joints freely in their cages without restriction and were observed daily for their general health condition, local infection, and mobility. The animals were sacrificed at 4 and 8 weeks postsurgery, and the knee joints were collected for an articular cartilage repair assessment.

### 2.6. Hematoxylin-Eosin Staining

Briefly, the samples were fixed in 4% paraformaldehyde for 24 hours. Subsequently, the samples were embedded in paraffin after decalcification and cut into 5 *μ*m sections with a microtome. Dewaxing and dehydration were performed with a xylene and ethanol aqueous solution, followed by HE staining by a conventional method. The sections were stained with hematoxylin for 5 minutes and eosin for 3 minutes.

### 2.7. Histological Grading

Histologic analysis of the repair tissue was performed for each specimen by two blinded observers using the Wakitani scoring system which is a well-detailed histologic grading system [13]. The scoring system is composed of five categories, including cell morphology, matrix staining, surface regularity, the thickness of the cartilage, and integration of donor with host, and assigns a score ranging from 0 to 14 points (Table 1).

### 2.8. Immunohistochemical Analysis

Endogenous peroxidase activity within the sections was quenched by incubating the sections with 3% H_2_O_2_ for 15 min after dewaxing and hydration. Then, the tissues were incubated with primary antibodies directed against type І collagen (ABP-COL-T1, American Biochemical & Pharmaceuticals), type II collagen (COL2A1, CAU23497, Biomatic), and MMP-13 (A-AJ1494a, Abgent). On the following day, the tissues were washed with PBS and incubated with secondary antibody antirabbit lgG (MaiXin Bio, China). In the negative controls, the primary antibody was replaced by PBS. They were counterstained with DAB (KT1009a, Abgent) and sealed with a glass slide by resin.

### 2.9. Glycosaminoglycan (GAG) Content

Sponge lysates obtained from the repair region after digestion for molecular biology were used to determine GAG content. This technique is based on a colorimetric assay using dimethylmethylene blue (DMB, Sigma) dye according to Goldberg's method [14]. The absorbance was measured at 525 nm with a spectrophotometer (Dynatech).

### 2.10. Biomechanical Testing

To assess the biomechanical properties of the cartilage, Young's elastic modulus was tested in six samples per group by the In Situ Nanomechanical Test System (Hysitron, USA). The radius of curvature of the conspherical diamond probe tip was 100 mm. Each indentation point uses a trapezoidal load function, with a loading time of 10 seconds, a holding time of 5 seconds, and an unloading time of 10 seconds. The cylindrical loading device was perpendicular to the repair area (RA) and moved forward at 200 nm/s.

### 2.11. Statistical Analysis

SPSS 19.0 (IBM, New York, USA) was applied to analyze all data. Differences among multiple groups were statistically analyzed using one-way ANOVA and post hoc comparisons (Dunnett's test). Values of *P* < 0.05 were considered statistically significant.

## 3. Results

### 3.1. The Identification of hUCB-MSCs and rBM-MSCs

**Figure 1:**
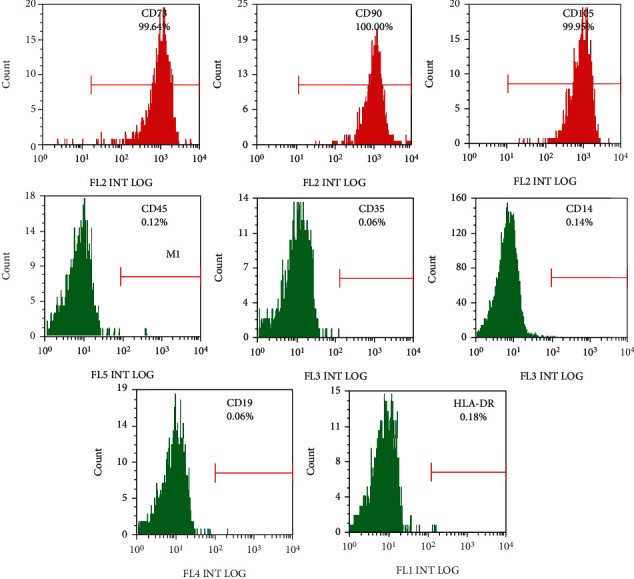
Flow-cytometric analysis of surface markers expressed on hUCB-MSCs and rBM-MSCs. hUCB-MSCs: human umbilical cord blood-derived mesenchymal stem cells; rBM-MSCs: rabbit bone marrow stem cells.

As presented in Figure 1, flow-cytometric data demonstrated that hUCB-MSCs and rBM-MSCs were positive for CD73, CD105, and CD90, but negative for CD45, CD34, CD14, CD19, and HLA-DR. Specifically, the positive rate of cell count was 99.64% for CD73, 100% for CD90, and 99.95% for CD105, respectively. These results demonstrated that the cultured cells have the biological characteristics of MSCs.

### 3.2. The Gross Assessment of Animal Knee Joints

After the surgery of animals, the hind limb of the operative side showed obvious swelling, but gradually subsided after 2 days. The incision of the operative site was healed well. All animals survived successfully, with no adverse events observed.

**Figure 2:**
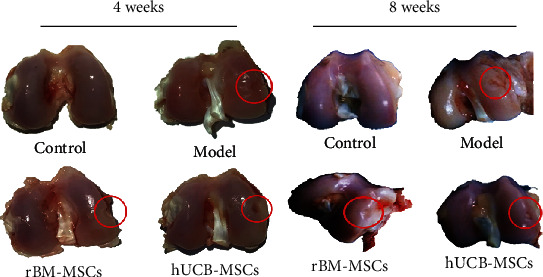
Macroscopic findings of the regenerating osteochondral defects on articular cartilage.

Four weeks after the treatment of the cartilage defect by implantation of hUCB-MSCs and rBM-MSCs, there were no effusion, adhesion, contracture, and other problems of the knees in both the hUCB-MSC and rBM-MSC groups. The defects of the cartilage were partially filled with undifferentiated tissue in both the hUCB-MSC and rBM-MSC groups, while no de novo repair tissue appeared in the model group and the tissue around the defect also began to be damaged and degenerate (Figure 2).

After 8 weeks, compared with the control group, the cartilage defect in the hUCB-MSC group significantly decreased, with about 1/2 of the previous defect and has a distinct boundary. In the rBM-MSC group, de novo repair tissue could be observed at the bottom of the cartilage defect, which accounts for about 1/3 of the defect area. However, there was still no de novo repair tissue in the model group (Figure 2).

#### 3.2.1. Histological Analyses

**Figure 3:**
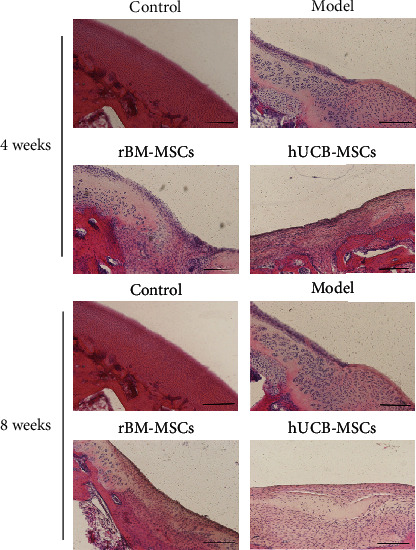
Microscopic findings of the regenerating osteochondral defects on articular cartilage (hematoxylin and eosin staining). Scale bar = 200 *μ*m.

Microscopically, 4 weeks after implantation of hUCB-MSCs, the de novo cells in the hUCB-MSCs group were flat, disordered, and tightly packed, with slightly shallow cytoplasm staining. However, only one layer of cells appeared on the surface of the defect area in the rBM-MSC group and model group (Figure 3).

After 8 weeks of transplantation, the de novo cells in the hUCB-MSCs group tended to be round and arranged loosely, but tended to be arranged in the normal way. The cytoplasm was shallowly stained, and the boundary between de novo cells and normal cells was obvious. In the rBM-MSCs group, the de novo cells are flat and tightly arranged and tend to be arranged in the form of normal cells. Besides, the cytoplasm is slightly stained. Unlike the hUCB-MSCs group, the boundary between de novo cells and normal cells in the group rBM-MSCs is not obvious. In the model group, only a layer of cells is formed on the surface of the defect area (Figure 3). To observe the repair situation of each group more intuitively, we conducted a quantitative analysis of tissue scoring. The results depicted that the hUCB-MSC group had the lowest score among the above groups, indicating the best repair of the defect area (Table 2).

#### 3.2.2. Immunohistochemical Analyses

**Figure 4:**
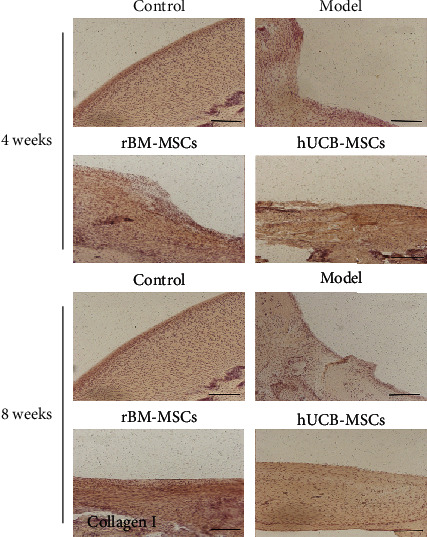
The changes in collagen I in the injured area of the cartilage after 4 or 8 weeks of cell transplantation. Scale bar = 200 *μ*m.

**Figure 5:**
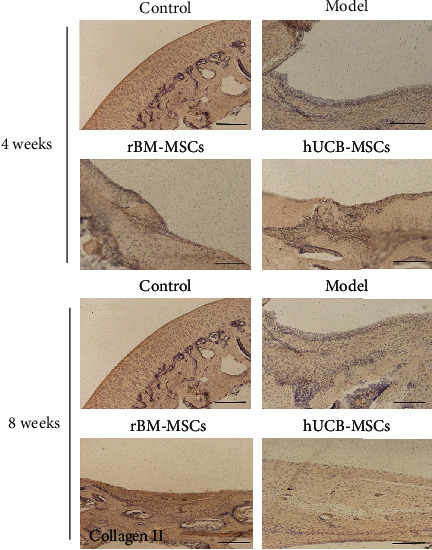
The changes in collagen II in the injured area of the cartilage after 4 or 8 weeks of cell transplantation. Scale bar = 200 *μ*m.

**Figure 6:**
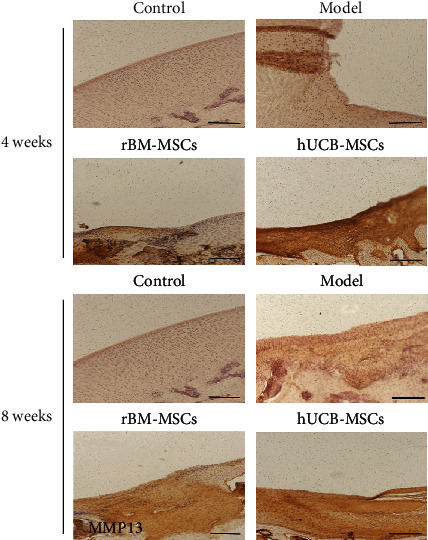
The changes in MMP-13 in the injured area of cartilage after 4 or 8 weeks of cell transplantation. MMP-13: matrix metalloproteinase-13. Scale bar = 200 *μ*m.

Immunohistochemistry revealed the differentiation of the repair tissue with positive staining for collagen I and II. Compared with the control group, the expression of collagen I was significantly higher in the hUCB-MSCs group, while the expression of collagen II was significantly lower, indicating the de novo tissue was composed of hyaline cartilage and fibrocartilage, and the content of fibrocartilage is higher. Compared with the control group, the expression of collagen I or II is similar to that of normal cartilage, indicating the de novo tissues in the rBM-MSCs group are similar to the normal tissues (Figures 4 and 5). In addition, MMP-13 in the de novo tissues of the hUCB-MSC and rBM-MSC groups was much higher than that of the normal group, suggesting that the de novo tissues had the risk of osteoarthritis (Figure 6).

#### 3.2.3. Glycosaminoglycan (GAG) Content

After 4 weeks of transplantation, the GAG content of the hUCB-MSCs group was 10 times that of the model group, and the rBM-MSCs group was 8 times that of the model group, but the hUCB-MSCs group with the highest content only reached 1/3 of the control group.

**Figure 7:**
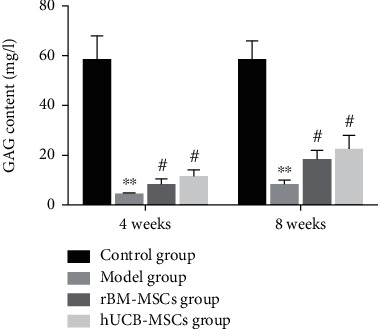
After 4 weeks or 8 weeks of cell transplantation, the changes of GAG content in cartilage injury area of different groups. ^∗^ Compared with the control group. ^#^Compared with the model group. GAG: glycosaminoglycan.

After 8 weeks of transplantation, GAG content in the hUCB-MSC group was 4.5 times higher than that in the model group and 3.5 times higher in the rBM-MSC group than that in the model group. Meanwhile, both the hUCB-MSC group and the rBM-MSC group were about 1/2 as high as those in the control group (Figure 7).

#### 3.2.4. Biomechanical Testing

**Figure 8:**
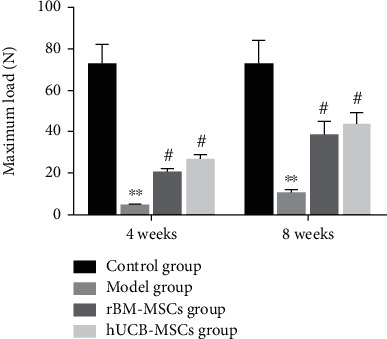
Quantitative analysis of articular cartilage repair (biomechanical evaluation). ^∗^Compared with the control group. ^#^Compared with the model group.

After 4 weeks of transplantation, the maximum load in the hUCB-MSCs group and the rBM-MSCs group was about twice that of the Model group, but much lower than that in the control group. After 8 weeks of transplantation, the maximum load of the hUCB-MSCs group was about 3 times that of the Model group, which was about the 2/5 of control group. The rBM-MSCs group was about twice that of the Model group and about 1/5 of the control group. In conclusion, our results indicated that there is a certain difference between the biomechanical properties of de novo tissues and normal tissues in the hUCB-MSCs group and rBM-MSCs group (Figure 8).

## 4. Discussion

Articular cartilage has limited and insufficient ability to self-regeneration once being damaged. To date, great efforts have been made; however, no treatment claimed to be effective can completely repair damaged articular cartilage. Potential therapies based on multidifferentiation characteristics of MSCs and for cartilage regeneration were widely studied. In addition, MSCs have a wide range of sources and can be obtained from a variety of mature organizations, such as bone marrow, adipose tissue, and synovium [15, 16]. Fetus tissues contain abundant MSCs [17], such as umbilical cord blood, placenta [18], and umbilical cord matrix [19]. Recently, hUCB-MSCs have been regarded as a more effective alternative source of cartilage regeneration. They can be collected noninvasively, which avoids ethical and technical problems, and can be stored in advance and quickly obtained when needed [20]. Besides, mesenchymal stem cells from hUCBs were more primitive than MSC isolated from other tissue sources [10, 21]. The higher proliferative capacity with a faster doubling time and consistent proliferation, even after 30 passages, might enable hUCBs as an alternative source of MSCs for clinical application. Moreover, the immune cells were immature and had low functional activity, which could not trigger the immune response and cause graft versus host disease [22]. Based on these properties, hUCB-MSCs were considered a promising alternative source of allogenic MSC. However, so far, few in vivo studies have been able to obtain reasonable evidence of cartilage regeneration using hUCB-MSCs. In the present study, we constructed a cartilage injury model and used rBM-MSCs as a positive control to explore the effect of hUCB-MSCs in cartilage repair. First, we identified the extracted cells and found that they were consistent with the phenotype of MSC [1]. Next, we observed the changes in the transplanted areas after the transplantation of the two types of cells at the macro- and microlevels. As expected, both the rBM-MSC group and hUCB-MSC group had good cartilage recovery ability, and the repair effect of the group hUCB-MSCs was better than that of the group rBM-MSCs.

Collagen is a protein family that forms the main body of a large molecular network that forms extracellular matrix (ECMs). So far, there are 28 known types of collagen. Among them, collagen I is the most collagen protein in the human body, accounting for 30% of the total protein [23]. Collagen I is parallel to the surface of the fibrous layer and is the main extracellular component of the layer, as well as an important marker of chondrocyte fibrosis [24]. The extracellular matrix of hyaline cartilage contains a variety of noncollagen and a variety of collagen. Among these collagens, collagen II is the main component. Besides, collagen II is essential for the structural integrity of tissue and is a marker of cartilage formation [25]. In this study, our data showed that after transplantation of hUCB-MSCs, the content of collagen І in the de novo tissues of the defect area was increased, while the content of collagen ІІ was slightly lower than that in the control group, indicating that the de novo tissues in the hUCB-MSCs group were a complex of fibrocartilage and hyaline cartilage, in which the content of fibrocartilage was slightly higher than that of clear cartilage. Conversely, after transplantation of rBM-MSCs, the expression of type І collagen and type ІІ collagen in neonatal tissue is closer to that in the normal group, indicating that the neonatal tissue in the bone marrow group is hyaline cartilage more similar to normal cartilage. MMP-13 is recognized as a key player in cartilage biology and OA pathology due to its ability to degrade type II collagen and a variety of other matrix components [26, 27]. Under pathological conditions, MMP-13 was expressed in sites where the extracellular matrix is overdegraded, such as OA, rheumatoid arthritis (RA), and various cancers [28]. Consistently, in our study, we found that MMP-13 in the new tissues of group hUCB-MSCs and group rBM-MSCs was much higher than that of the control group, suggesting that the new tissues had the risk of forming OA.

In view of the fact that both hUCB-MSCs and rBM-MSCs can promote cartilage tissue repair, we then evaluated the biochemical composition and biomechanics of the de no repair tissue. Glycosaminoglycans (GAG) are essential for life as they are responsible for orchestrating many essential functions in development and tissue homeostasis, including biophysical properties and roles in cell signaling and extracellular matrix assembly [29]. Besides, GAGs have been incorporated into biomaterials for use in tissue engineering, drug delivery, and regenerative medicine purposes. Previously, it was reported that GAG is an important component in the ECM of cartilage tissue, which is also active in promoting chondrogenesis [23]. In the presented study, the content of GAG in the de novo tissues of the groups of hUCB-MSCs and rBM-MSCs was significantly increased, but it was still lower than that of the control group. The mechanical properties of tissue engineering are also a major terminal for the regeneration of many biological tissues. Therefore, we also conducted a biomechanical analysis. The results indicated that both the hUCB-MSC group and the rBM-MSC group have the biomechanical function of tissue repair, and the hUCB-MSCs group was better than the rBM-MSCs group, but there is still a certain gap with the normal group.

## 5. Conclusion

Both the hUCB-MSCs and rBM-MSCs could repair the cartilage injury to a certain extent, among which the hUCB-MSCs group repaired quickly. However, there are some limits in our study. First, although we have demonstrated the effect of hUCB-MSCs on cartilage injury, we did not conduct further dose study on hUCB-MSCs. Therefore, next, we extracted exosomes from the cells to compare the two cellular effects of intact hUCB-MSC and hUCB-MSC-derived secretions. Second, the expression of MMP and secreted ECM component is extremely relevant; however, the expression changes in our manuscript were detected only by immunohistochemistry. Next, western blot was used to further detect the expression of the above-related proteins.

## Figures and Tables

**Table 1:** Histological grading scale for the defects of the cartilage (Wakitani et al. [13]).

Category	Points
Cell morphology	
Hyaline cartilage	0
Mostly hyaline cartilage	1
Mostly fibrocartilage	2
Mostly noncartilage	3
Noncartilage only	4
Matrix-staining (metachromasia)	
Normal (compared with the host adjacent cartilage)	0
Slightly reduced	1
Markedly reduced	2
No metachromatic stain	3
Surface regularity^a^	
Smooth (>3/4)	0
Moderate (>112-314)	1
Irregular (1/41/2)	2
Severely irregular (<1/4)	3
Thickness of cartilage^b^	
>2/3	0
1/3-2/3	1
<1/3	2
Integration of donor with host adjacent cartilage	
Both edges integrated	0
One edge integrated	1
Neither edge integrated	2
Total maximum	14

^a^Total smooth area of the reparative cartilage compared with the entire area of the cartilage defect. ^b^Average thickness of the reparative cartilage compared with that of the surrounding cartilage.

**Table 2:** Histological score of the defect area.

Cycle	hUCB-MSCs	rBM-MSCs	Model
4 weeks	11	13	14
8 weeks	7	10	14

hUCB-MSCs: human umbilical cord blood-derived mesenchymal stem cells; rBM-MSCs: rabbit bone marrow stem cells.

## Data Availability

The data used to support the findings of this study are available from the corresponding author upon request.

## References

[1] Neybecker P., Henrionnet C., Pape E. (2018). In vitro and in vivo potentialities for cartilage repair from human advanced knee osteoarthritis synovial fluid-derived mesenchymal stem cells. *Stem Cell Research & Therapy*.

[2] Bae D. K., Yoon K. H., Song S. J. (2006). Cartilage healing after microfracture in osteoarthritic knees. *Arthroscopy*.

[3] Park Y. B., Ha C. W., Kim J. A. (2017). Single-stage cell-based cartilage repair in a rabbit model: cell tracking and in vivo chondrogenesis of human umbilical cord blood-derived mesenchymal stem cells and hyaluronic acid hydrogel composite. *Osteoarthritis and Cartilage*.

[4] Mobasheri A., Csaki C., Clutterbuck A. L., Rahmanzadeh M., Shakibaei M. (2009). Mesenchymal stem cells in connective tissue engineering and regenerative medicine: applications in cartilage repair and osteoarthritis therapy. *Histology and Histopathology*.

[5] Chamberlain G., Fox J., Ashton B., Middleton J. (2007). Concise review: mesenchymal stem cells: their phenotype, differentiation capacity, immunological features, and potential for homing. *Stem Cells*.

[6] Chang Y. H., Liu H. W., Wu K. C., Ding D. C. (2016). Mesenchymal stem cells and their clinical applications in osteoarthritis. *Cell Transplantation*.

[7] Wu K. C., Chang Y. H., Liu H. W., Ding D. C. (2019). Transplanting human umbilical cord mesenchymal stem cells and hyaluronate hydrogel repairs cartilage of osteoarthritis in the minipig model. *Ci Ji Yi Xue Za Zhi*.

[8] Lindenmair A., Hatlapatka T., Kollwig G. (2012). Mesenchymal stem or stromal cells from amnion and umbilical cord tissue and their potential for clinical applications. *Cell*.

[9] Pereira R. C., Costa-Pinto A. R., Frias A. M., Neves N. M., Azevedo H. S., Reis R. L. (2017). In vitro chondrogenic commitment of human Wharton's jelly stem cells by co-culture with human articular chondrocytes. *Journal of Tissue Engineering and Regenerative Medicine*.

[10] Ha C. W., Park Y. B., Chung J. Y., Park Y. G. (2015). Cartilage repair using composites of human umbilical cord blood-derived mesenchymal stem cells and hyaluronic acid hydrogel in a minipig model. *Stem Cells Translational Medicine*.

[11] Fisher M. B., Belkin N. S., Milby A. H. (2015). Cartilage repair and subchondral bone remodeling in response to focal lesions in a mini-pig model: implications for tissue engineering. *Tissue Engineering. Part A*.

[12] Zhang Y., Liu S., Guo W. (2018). Human umbilical cord Wharton's jelly mesenchymal stem cells combined with an acellular cartilage extracellular matrix scaffold improve cartilage repair compared with microfracture in a caprine model. *Osteoarthritis and Cartilage*.

[13] Wakitani S., Goto T., Pineda S. J. (1994). Mesenchymal cell-based repair of large, full-thickness defects of articular cartilage. *The Journal of Bone and Joint Surgery. American Volume*.

[14] Goldberg R. L., Kolibas L. M. (1990). An improved method for determining proteoglycans synthesized by chondrocytes in culture. *Connective Tissue Research*.

[15] Zuk P. A., Zhu M., Mizuno H. (2001). Multilineage cells from human adipose tissue: implications for cell-based therapies. *Tissue Engineering*.

[16] de Sousa E. B., Casado P. L., Moura Neto V., Duarte M. E., Aguiar D. P. (2014). Synovial fluid and synovial membrane mesenchymal stem cells: latest discoveries and therapeutic perspectives. *Stem Cell Research & Therapy*.

[17] Campagnoli C., Roberts I. A., Kumar S., Bennett P. R., Bellantuono I., Fisk N. M. (2001). Identification of mesenchymal stem/progenitor cells in human first-trimester fetal blood, liver, and bone marrow. *Blood*.

[18] Park Y. B., Seo S., Kim J. A., Heo J. C., Lim Y. C., Ha C. W. (2015). Effect of chondrocyte-derived early extracellular matrix on chondrogenesis of placenta-derived mesenchymal stem cells. *Biomedical Materials*.

[19] Wang H. S., Hung S. C., Peng S. T. (2004). Mesenchymal stem cells in the Wharton's jelly of the human umbilical cord. *Stem Cells*.

[20] Malgieri A., Kantzari E., Patrizi M. P., Gambardella S. (2010). Bone marrow and umbilical cord blood human mesenchymal stem cells: state of the art. *International Journal of Clinical and Experimental Medicine*.

[21] Can A., Karahuseyinoglu S. (2007). Concise review: human umbilical cord stroma with regard to the source of fetus-derived stem cells. *Stem Cells*.

[22] Lu L. L., Liu Y. J., Yang S. G. (2006). Isolation and characterization of human umbilical cord mesenchymal stem cells with hematopoiesis-supportive function and other potentials. *Haematologica*.

[23] Rittié L., Rittié L. (2017). Type I Collagen purification from rat tail tendons. *Fibrosis. Methods in Molecular Biology, vol 1627*.

[24] Athanasiou K. A., Almarza A. J., Detamore M. S., Kalpakci K. N. (2009). Tissue engineering of temporomandibular joint cartilage. *Synthesis Lectures on Tissue Engineering*.

[25] Orajarvi M., Laaksonen S., Hauru R. (2018). Changes in type I and type II collagen expression in rat mandibular condylar cartilage associated with aging and dietary loading. *Journal of Oral & Facial Pain and Headache*.

[26] Otero M., Plumb D. A., Tsuchimochi K. (2012). E74-like factor 3 (ELF3) impacts on matrix metalloproteinase 13 (MMP13) transcriptional control in articular chondrocytes under proinflammatory stress. *The Journal of Biological Chemistry*.

[27] Xu M., Zhang L., Zhao L. (2013). Phosphorylation of osteopontin in osteoarthritis degenerative cartilage and its effect on matrix metalloprotease 13. *Rheumatology International*.

[28] Fan Z., Tardif G., Boileau C. (2006). Identification in human osteoarthritic chondrocytes of proteins binding to the novel regulatory site AGRE in the human matrix metalloprotease 13 proximal promoter. *Arthritis and Rheumatism*.

[29] Rnjak-Kovacina J., Tang F., Whitelock J. M., Lord M. S. (2018). Glycosaminoglycan and proteoglycan-based biomaterials: current trends and future perspectives. *Advanced Healthcare Materials*.

