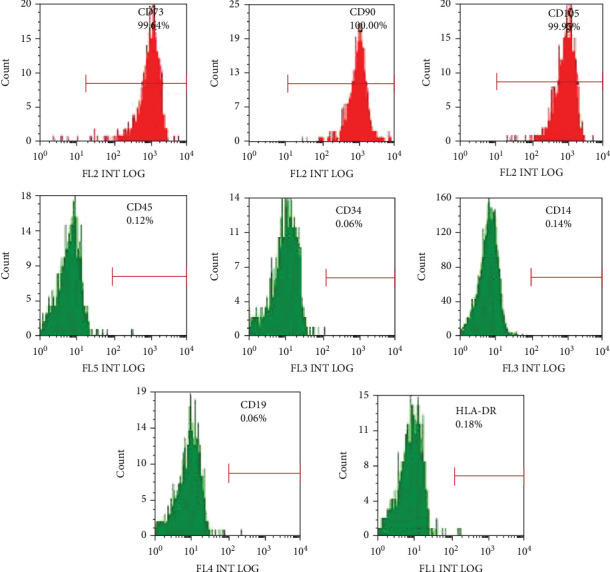# Corrigendum to “Transplantation of Human Umbilical Cord Blood-Derived Mesenchymal Stem Cells Improves Cartilage Repair in a Rabbit Model”

**DOI:** 10.1155/2025/9780636

**Published:** 2025-06-25

G. Yang, J. Shao, J. Lin, et al., “Transplantation of Human Umbilical Cord Blood-Derived Mesenchymal Stem Cells Improves Cartilage Repair in a Rabbit Model”, *BioMed Research International*, no. 1 (2021): 1-32, https://doi.org/10.1155/2021/6380141

In the article, the authors wish to clarify the inconsistent descriptions of hUCB-MSCs and hUC-MSC in the paper. Human umbilical cord-derived mesenchymal stem cells (hUC-MSCs) should be used throughout the article, not human umbilical cord blood-derived mesenchymal stem cells (hUCB-MSCs). The title of the article has been updated in the article to reflect this as follows:

“Transplantation of Human Umbilical Cord-Derived Mesenchymal Stem Cells Improves Cartilage Repair in a Rabbit Model”.

Additionally, the Section 2.1, “Isolation and Culture of hUC-MSCs” should be replaced with the below:

“Human umbilical cords were collected from full-term healthy infants. Informed consent was obtained according to the institutional guidelines. About 8-cm-long maternal umbilical cord was separated under sterile environment and soaked in sterile PBS immediately. Blood vessels and other tissues of the umbilical cords were removed, and Wharton's jelly was cut into 1–3-mm three small pieces and adhered on the bottom of the culture dishes, and then added with complete culture medium (MesenGro hMSC Medium, StemRD). Half medium has been changed every 3 days. Pieces were removed after 7 days of culture, and the medium was replaced. After about 14 days, hUC-MSCs were observed growing into a dense group, and then it has been passaged with 0.25% trypsin–EDTA (Sigma-Aldrich) when reached 80%–90% fusion.”

Lastly, Figure 1 has been revised as follows, correcting CD35 to CD34.

The authors apologize for this error.

## Figures and Tables

**Figure 1:**